# Cortical spreading depolarizations in patients with intracerebral hemorrhage: preliminary data

**DOI:** 10.1186/cc14548

**Published:** 2015-03-16

**Authors:** AJ Schiefecker, R Beer, M Kofler, B Pfausler, I Unterberger, P Lackner, G Broessner, P Rhomberg, F Sohm, M Mulino, C Thome, M Fabricius, E Schmutzhard, R Helbok

**Affiliations:** 1 Medical University of Innsbruck, Austria; 2 Rigshospitalet, Copenhagen, Denmark

## Introduction

Perihematomal edema (PHE) expansion contributes to increased morbidity and mortality after spontaneous intracerebral hemorrhage (ICH). Pathophysiology of PHE is incompletely understood. Recently, the role of cortical spreading depolarizations (CSDs) in secondary brain injury was established in subarachnoid and traumatic brain injury patients. However, the value of CSDs after ICH is not known.

## Methods

Patients with ICH fulfilling the inclusion criteria were prospectively enrolled in the observational COSBID study (Co-operative Study on Brain Injury Depolarisations). g.BSamp (g.tec, Austria) connected to PowerLab and LabChart software (Adinstruments) was used for electrocorticography (EcoG). Electrocardiogram patches at the patient's shoulder and bed served as groundings, and a surface reference electrode was glued on the mastoid. The duration of EcoG depressions was defined as the time between depression onset and start of EcoG recovery in the integral of power calculations (0.5 to 45 Hz; 60 seconds time constant decay). Brain tissue oxygen tension (PbtO_2_), cerebral blood flow (CBF), cerebral metabolism and intracranial pressure were monitored in the PHE region. Data are presented as median and interquartile range.

## Results

Eighteen patients with ICH (ICH volume: 54 (33 to 69) ml) were analyzed. Hematoma evacuation was performed in 17 patients, one subject underwent craniectomy only. Patients were 60 (55 to 67) years old and 38% female. Monitoring time per patient was 10 (6 to 14) days. A total of 129 CSDs with 16 (10 to 29) minutes of EcoG depression were observed. Eighty-four percent (*n *= 15) of patients showed expansion of PHE by 25 (10 to 50) ml within 3 to 6 days after bleeding. Neuromonitoring probes were 35 (23 to 58) mm distant from the EcoG strip. CSDs occurred in 73% (*n *= 11) of patients with PHE expansion. The interval between CSDs was 98 minutes (25 to 308). CSDs were associated with a significant decrease of PbtO_2_ (-4 mmHg (-3; -7); duration 10 (5 to 23) minutes) in 68% (52/77), CBF changes in 95% (19/20) and metabolic derangement in 80% (80/100) of CSDs. PHE expansion was observed in all patients with spreading convulsions (*n *= 2) and patients with repetitive CSDs occurring as clusters (*n *= 3).

## Conclusion

CSDs are common in ICH patients and associated with perihematomal PbtO_2_ decreases and metabolic derangement. Especially, clusters of CSDs might be associated with detrimental metabolic changes of the perihematomal brain tissue.

